# Sir Astley Cooper, His Life, Character, and Writings

**Published:** 1841-04

**Authors:** 


### OBITUARY.

### SIR ASTLEY COOPER,—HIS LIFE, CHARACTER, AND WRITINGS.*

Sir Astley Cooper was the fourth son of the Rev. Dr. Samuel Cooper, of Yarmouth, in the county of Norfolk. His mother was the daughter of Mr. James Bransby, of Shottisham, a co-heiress descended from the family of Paston, Earls of Yarmouth, a lady distinguished by high intellectual attainments, and known as the author of a work of fiction called "The Exemplary Mother." Astley Cooper was born at Brooke, in Norfolk, on the 23d of August, 1768, where he remained till the age of fourteen, receiving his rudimental education at the village school, and the higher branches of learning from his father and the Rev. Joseph Harrison, a distinguished classic. His mind was early directed to the study of surgery, and he was, when about fifteen years of age, placed with Mr. Turner, who was at that time a general practitioner at Yarmouth. Here he only remained a few months, when he came to London, and was apprenticed to Mr. William Cooper, his uncle, then one of the surgeons to Guy's Hospital. Shortly afterwards, at his own desire, he was transferred to Mr. Cline, at St. Thomas's Hospital. The early part of his pupilage was not marked by that unremitting industry which afterwards distinguished him; and it was not until after he had spent a short time in Edinburgh in 1787, and had been appointed demonstrator of anatomy at St. Thomas's Hospital, under Mr. Cline, that his great natural powers were called forth, and matured by the utmost industry in the dissecting-room and the wards of the hospital. In 1791 he began to give a portion of the anatomical course in conjunction with Mr. Cline. At this time no distinct courses of lectures on the principles and practice of surgery were given in London, the maxims of the day being included in the anatomical course; but Mr. Cooper, with the concurrence of the surgeons of Guy's and St. Thomas's, commenced the lectures, since so well known by the publication of repeated editions, and which very soon became the most popular of the day. Towards the close of 1791 he married Miss Cock, of Tottenham, a relative of Mr. Cline. In 1792 he went to Paris, and attended the practice and lectures of Dessault and Chopart. He commenced practice in the same year, residing for six years in Jeffrey's Square, St. Mary's Axe. He then removed to New Broad Street, where he remained until the year 1815, when he removed to the West-end, having been surgeon to Guy's Hospital since 1800. He lived in the house now occupied by Mr. Bransby Cooper, 2, New Street, Spring Gardens, and

* From No. 22 of the Provincial Medical and Surgical Journal.

enjoyed an immense practice until 1827, when he retired into the country; but it soon appeared that he was not formed for a life of "inglorious ease," and he shortly afterwards returned to London, and resided until his death in Conduit Street. Here he gave a series of professional *soirées*, which were attended by most of the practitioners in London; but he never regained a tithe of the practice he had formerly had. He was appointed surgeon to George IV., and in 1821 was created a baronet, with remainder, in default of male issue, to Astley Paston, the fourth son of his second brother, the Rev. Dr. Samuel Cooper, rector of Ingoldsthorpe and Barton, Norfolk. He continued his lectures at Guy's Hospital until 1826, in which year he became president of the College of Surgeons; and in 1827 he was appointed serjeant-surgeon to George IV. Lady Cooper died in the same year, and in 1828 Sir Astley married the daughter of Mr. Jones, of Derry Ormond, Cardiganshire. He was again elected president of the College of Surgeons in 1837, continuing his practice and pathological labours until his last illness. With the exception of occasional attacks of gout and vertigo, he invariably enjoyed good health, until about six weeks since, when he was walking to church at Strathfieldsaye with his Grace the Duke of Wellington, and was seized with violent and irregular action of the heart, accompanied by considerable difficulty of breathing. He returned to town, and immediately put himself under the care of Dr. Bright and Mr. Bransby Cooper, when it became evident that effusion to some extent had taken place into the pericardium. He was occasionally delirious, and though not suffering acutely, was troubled by frequent cough and dyspnœa. Effusion now came on in the left pleura, and Dr. Chambers was consulted, when elaterium was given in tolerably free doses; but he was beyond the power of medicine, and died without a struggle about one o'clock on Friday, February 12th, in the seventy-third year of his age. A post-mortem examination was made of the body, when it appeared that the heart was enlarged, and its walls diminished in thickness. There was serous effusion in the pericardial and pleural cavities, and several patches of atheromatous deposits upon or between the coats of the aorta. His title, with the bulk of his landed property, descends to the present baronet, a younger brother of Mr. Bransby Cooper, who has a large family. His extensive and valuable museum, with his various works, pass to Mr. Bransby Cooper.

Besides the baronetcy, the late Sir Astley possessed several other marks of distinction. Louis Philippe conferred on him the cross of the legion of honour; he was elected corresponding member of the National Institute of France, and of most of the learned societies of Germany and America; and from William IV. he received the distinction of grand cross of the royal Guelphic order. He was also a doctor of civil law of the University of Oxford. His professional income was probably greater than that of any other surgeon or physician of our own or former days, in one year exceeding 21,000*l.*, and for several years averaging 15,000*l.* He has more than once received 1000*l.* for a single operation, and has been confidentially employed by their late majesties George IV. and William IV., his Royal Highness the Duke of York, and all the leading aristocracy of the country, in addition to numerous eminent individuals from every quarter of the globe.

" ——— Sidera terræ

Ut distant, ut flamma mari, sic utile recto,"

says Lucan; but it is only necessary to refer to the character of Sir Astley Cooper to prove the fallacy of this celebrated aphorism. His success, though of course dependent in some degree on the fortunate circumstances which surrounded the commencement of his professional career, was otherwise entirely dependent on his own merits and exertions. He was never guilty of open intrigue or secret inuendo against the character of a professional brother, never attempting to build his own reputation on the ruins of that of another. He was always ready to afford his advice and assistance to the junior members of the profession, hundreds of whom now living cherish his name with affectionate veneration. As a public lecturer he was probably the most popular that ever appeared in an English school; an enthusiast in his profession, and eloquent in its praise, he poured forth the treasures of his clear understanding with an energy, frankness, and affability, that, combined with his liberal feelings and engaging countenance, completely fascinated his auditors. His class for some years exceeded four hundred in number; and we never yet saw a man who had heard him who was not loud in his praise, and grateful for his instructions. As an hospital-surgeon he was distinguished for the amenity of his deportment, his ready tact in the discrimination of disease, and the no less subtle ingenuity in forming plans for its cure. As an operator, though never particularly neat, he was always expert, rapid, and unusually successful. His industry is probably without a parallel in the history of the art. He was always a very early riser; and even at the time when he was employed from six in the morning till midnight in attendance on his public and private patients, with the duties of his lectureship, he would spend great part of many nights in anatomical and pathological pursuits, as is proved by the statements of his friends, and the fact that his most laborious works appeared while he was in the zenith of his popularity. Even when past the usual age of mental activity, when he had reaped the "otium cum dignitate," so far from luxuriating in the ease of mental repose, he composed his last work on the Anatomy of the Breast, which is founded on upwards of two hundred and fifty preparations in his private museum. During the period he was constantly employed, he maintained the habit of noting down all interesting occurrences in his case-book, which are preserved from 1794 to his latest days, affording the most valuable materials for the illustration of surgical precepts.

Let us now turn to the character of Sir Astley as a scientific man, and consider for a moment the discoveries which have alike established his own fame, advanced the science he professed, and increased the resources of the working surgeon in the daily exercise of his art. The mere enumeration of these is all that is necessary to place the name of Sir Astley high among the most renowned benefactors of humanity, and to afford on the slightest knowledge of the comparative state of surgery fifty years ago and at the present day, the most startling conviction of the immense influence which may be exerted over a class or a nation by the labours and talents of a single individual. It is not half a century since it was doubted in our schools whether the hip-joint was ever dislocated; and those who admitted the possibility of the occurrence, doubted the practicability of its reduction. Cases were constantly met with in the hospitals where dislocations had been treated as fractures until the period had passed in which reduction could be effected; and others, perhaps equally numerous, in which irreparable injury had been inflicted by pulling a fractured limb, under the belief that it was dislocated. Sir Astley cleared up this cloud of ignorance and error; and now, as a result of his researches, almost every fracture and dislocation is readily recognized by the merest tyro, and their treatment rendered more simple and efficacious.

We turn to hernia, and trace similar improvements to the same source. The various species of hernia have been distinguished from each other, and from the different diseases with which they had been, or might be, confounded. The anatomy of the parts through which the intestine might protrude, and its various coverings after protrusion, were carefully investigated, with the effect of rendering our knowledge of the descent far more precise, increasing our means of preventing strangulation, and making the operation after strangulation had occurred far more safe and effectual.

The experiments of Sir Astley on the ligature of arteries, and the collateral circulation subsequently set up, followed by his bold, but strictly warrantable, operations on the carotid and aorta, had an equally remarkable effect on the surgical therapeutics of aneurism. He was the first to demonstrate the practicability and safety of tying the carotid in the living subject; and it is fair to conclude that this operation has been the means not only of curing diseases otherwise fatal, but that it has led to a far more philosophical view of the treatment of aneurism than was received in the time of Hunter. He was the first to tie the aorta; and subsequent facts have shown that this will, in all probability, confer on the surgeon the power of directly saving life in some cases.

The work of Sir Astley on the Anatomy and Diseases of the Testis affords another example of the effect of his labours in advancing surgical science; various diseases being distinguished from malignant growths and depositions, and made readily curable, for which the sufferers were formerly doomed to castration. Indeed, there is scarcely a department of surgery, the practice of which has not been vastly improved by his unwearied industry and practical tact. He was the first to remedy obstruction in the Eustachian tube by puncturing the membrana tympani; the first to let off the fluid of spina bifida by repeated punctures, and thereby cure a disease previously considered beyond the reach of art. He was the first who cut into the membranous portion of the urethra through the perinæum, rather than puncture the bladder, either above the pubes or through the rectum; the first who practised the removal of exostoses by paring off their investing periosteum, thereby removing the medium of their nutrition, causing their death, and exfoliation. His latest work, on the Anatomy of the Breast, is one which will always be the standard authority on the subject, and is only equalled by his volume on the Non-malignant Diseases of the Breast, a work which is distinguished for the diagnosis it contains of the simple from the malignant diseases, and the sound precepts laid down as to the tumours which might be removed without a fear of their return,—points of the first interest to the practical man.

We have purposely abstained from filling our pages with the various anecdotes which have been going the round of the daily and weekly periodicals, knowing many of them to be apocryphal and others decidedly false, and have now merely to add a list of the different contributions for which the profession is indebted to Sir Astley, all of which we hope to see published in a collected form with the life of their author, under the able editorship of his nephew. In answer to some illiberal attacks in the "Chronicle," ascribing *avarice* to Sir Astley, we have only to state that, in addition to his well-known generosity during life, he has freely endowed a perpetual studentship, to be in the gift of the College of Surgeons, as a lasting memento of his devotion to the science he professed.

For the following list of the various published writings of Sir Astley Cooper we are chiefly indebted to the memoir which appeared in Mr. Pettigrew's "Medical Portrait Gallery."

1798. Two papers in the "Medical Researches;" one on a case of diaphragmatic hernia; the other detailing three instances of obstruction to the thoracic duct, and showing, by dissection, the channels through which nutrition was carried on.

1800. A paper in the "Philosophical Transactions," entitled, "Observations on the Effects which take place from the Destruction of the Membrana Tympani of the Ear."

1801. In the "Philosophical Transactions," an "Account of an Operation for the Removal of a particular species of Deafness." For this paper he obtained the Copley medal of the Royal Society.

1804. His work on hernia appeared, entitled, "The Anatomy and Surgical Treatment of Inguinal and Congenital Hernia." A second edition was brought out in 1827.

1805. In the Edinburgh Medical and Surgical Journal we find a "Case of Malformation of the Genito-urinary Organs."

In the Transactions of the Medico-Chirurgical Society, we find—

1809. Vol. I. Two cases in which the carotid artery was tied, once successfully.

1812. Vol. II. "Dissection of a Limb on which the Operation for Popliteal Aneurism had been performed." Also, "Some Observations on Spina Bifida."

1813. Vol. IV. "History of a Case of Premature Puberty," and "An Account of the Anastomoses of the Arteries of the Groin."

1815. Vol. VIII. "Three Cases of Calculi removed from the Bladder without the use of cutting Instruments." This paper was written to show the extreme dilatibility of the female urethra.

1817. Vol. XI. "Account of a Case in which numerous Calculi were extracted from the Urinary Bladder of the Male without the employment of cutting Instruments."

1818. Vol. XII. Contains the history of a case in which a fatty tumour, weighing upwards of thirty-seven pounds, and measuring eighteen inches around its neck, was removed from the walls of the abdomen, the patient completely recovering.

1818—20. The surgical essays of Sir Astley and Mr. Travers appeared, those of the former being—1. On Dislocations. 2. The well-known Case of Ligature of the Aorta. 3. On Exostoses. 4. On Dislocations and Fracture of the Hip and Knee-joint. 5. On unnatural Apertures in the Urethra. 6. On Encysted Tumours. We have alluded to all these with the exception of the last, which is written to prove that encysted tumours take their origin in obstruction and enlargement of the sebaceous follicles.

1822. His great work appeared, "A Treatise on Dislocations and Fractures of the Joints;" and in

1823. An Appendix was added, on fractures of the neck of the thigh-bone, in consequence of the strictures of Mr. Earle.

1829. We have "Illustrations of the Diseases of the Breast. Part I." And in

1830. "Observations on the Structure and Diseases of the Testis."

1832. "The Anatomy of the Thymus Gland."

1836. In the first number of the Guy's Hospital Reports there are papers on the anastomoses of the femoral and inguinal vessels, and an account of the *post-mortem* examination in 1821 of the patient whose carotid artery he had tied in 1808. In the subsequent numbers we have a minute dissection of an unusually formed placenta and imperfect fœtus, and some observations on the thyroid gland contained in a paper by Mr. King. Also, "Some Experiments and Observations on Tying the Carotid and Vertebral Arteries, and the Pneumogastric, Phrenic, and Sympathetic Nerves." "On Spermatocele, or Varicocele of the Spermatic Cord." "On Dislocation of the Os Humeri upon the Dorsum Scapulæ, and upon Fractures near the Shoulder-joint." And the last paper he ever published, which was in the number for October, 1840, the "Dissection of a supposed Hermaphrodite."

His work "On the Anatomy of the Breast," appeared in 1839. His "Lectures on the Theory and Practice of Surgery" have gone through various editions. The only authorised edition is that of Mr. Tyrrell, of which only three volumes have appeared, in 1824, 1825, and 1827.

Sir Astley was engaged until his death in the completion of other publications and the correction of his older ones; and we understand that most of his papers are in a state which will render their arrangement far from difficult; and it is to be hoped that the public will shortly be favoured with the completion of his work on the Breast, which will comprise the various malignant diseases of this organ.

---

